# Sound-seeking before and after hearing loss in mice

**DOI:** 10.1038/s41598-024-67577-7

**Published:** 2024-08-19

**Authors:** Jessica Mai, Rowan Gargiullo, Megan Zheng, Valentina Esho, Osama E. Hussein, Eliana Pollay, Cedric Bowe, Lucas M. Williamson, Abigail F. McElroy, Jonny L. Saunders, William N. Goolsby, Kaitlyn A. Brooks, Chris C. Rodgers

**Affiliations:** 1 Department of Neurosurgery, Emory University School of Medicine, Atlanta, GA 30322 USA; 2 Neuroscience Graduate Program, Emory University, Atlanta, GA 30322 USA; 3 Department of Cell Biology, Emory University School of Medicine, Atlanta, GA 30322 USA; 4 Department of Otolaryngology-Head and Neck Surgery, Emory University School of Medicine, Atlanta, GA 30308 USA; 5 Department of Biomedical Engineering, Georgia Tech and Emory University School of Medicine, Atlanta, GA 30322 USA; 6 Department of Biology, Emory College of Arts and Sciences, Atlanta, GA 30322 USA; 7 Department of Neurology, University of California, Los Angeles, Los Angeles, CA 90095 USA

**Keywords:** Auditory system, Sensorimotor processing, Experimental models of disease

## Abstract

How we move our bodies affects how we perceive sound. For instance, head movements help us to better localize the source of a sound and to compensate for asymmetric hearing loss. However, many auditory experiments are designed to restrict head and body movements. To study the role of movement in hearing, we developed a behavioral task called sound-seeking that rewarded freely moving mice for tracking down an ongoing sound source. Over the course of learning, mice more efficiently navigated to the sound. Next, we asked how sound-seeking was affected by hearing loss induced by surgical removal of the malleus from the middle ear. After bilateral hearing loss sound-seeking performance drastically declined and did not recover. In striking contrast, after unilateral hearing loss mice were only transiently impaired and then recovered their sound-seek ability over about a week. Throughout recovery, unilateral mice increasingly relied on a movement strategy of sequentially checking potential locations for the sound source. In contrast, the startle reflex (an innate auditory behavior) was preserved after unilateral hearing loss and abolished by bilateral hearing loss without recovery over time. In sum, mice compensate with body movement for permanent unilateral damage to the peripheral auditory system. Looking forward, this paradigm provides an opportunity to examine how movement enhances perception and enables resilient adaptation to sensory disorders.

## Introduction

Humans and other animals explore the world through body movement. For example, we process visual scenes by scanning our eyes over them^1^ and we recognize physical objects by running our hands and fingers over them^2,3^. In a similar vein, mice coordinate sniffing with locomotion to track down scents^4^, vision with head movements to gauge distances^5,6^, and whisker movement with touch to recognize objects^7,8^. To contend with the active nature of sensation, experimentalists may attempt to isolate the cognitive processes of sensory processing and motor control by physically restraining the subject (e.g., with head fixation) or by using experimental designs that enforce a temporal separation in sensation, deliberation, and motor response^9^. While separating sensation and action in this way has indeed illuminated fundamental principles of decision-making^10^, our ability to extrapolate from restrained to free behavior is ineluctably limited. Moreover, the ubiquity of neural signals about body movement in sensory brain areas even in head-fixed animals^11–13^ (but see ref^14^) suggests that physical restraint may not effectively isolate sensory processing from movement signals after all.

Although active sensing has primarily been studied in the modalities of vision, touch, echolocation, and electrosensation, hearing is also an active sensorimotor process^15^. Species with limited ability to move the ears independently can still move the head to optimize the collection of auditory information^16–22^. For instance, we might turn and face a person in order to better hear them. Head movements can be used to favor the better ear in noisy environments like schoolrooms^23^ or to adapt to hearing loss^24–26^.

Neuroscientists often treat body movement as a confound whose effects must be canceled out of sensory input by dedicated circuitry throughout the neuraxis^27–30^. However, body movement can also create an opportunity to *enhance* sensory processing through neural circuits and computations that are less well understood^31,32^. These circuits and computations are difficult to study with only the tools available for human subjects. Using mice as a model system enables a greater range of tools, such as cell type-specific monitoring and manipulation. Moreover, mice have excellent hearing, which they must integrate with movement in order to survive and thrive. For instance, parent mice seek out their lost pups by the sound of their cries and bring them back to the nest^33–35^. Like humans, mice are “champion generalists”^36^ who rely on their adaptability to survive and thrive in varied and challenging environments.

In this project, we set out to understand how mice actively seek out sound and how they adapt to hearing loss. We developed a behavioral paradigm named sound-seeking, in which mice explore a multi-chambered arena to track down an ongoing sound source. Next, we surgically induced either unilateral or bilateral hearing loss in order to determine whether and how mice could compensate for disruption of peripheral input. After bilateral hearing loss, mice performed poorly and resorted to exhaustive search. In contrast, mice with unilateral hearing loss gradually regained good performance and relied increasingly on an active strategy of sequentially sampling each chamber for sound. The effect of hearing loss on the acoustic startle reflex was stable over time, indicating that the impairment and recovery we observed in sound-seeking was unlikely to be due to global changes in auditory sensitivity. Looking forward, these results lay the groundwork for understanding how sensory and motor neural circuits enable successful navigation of complex auditory environments and recovery from sensory impairment.

## Results

### The sound-seeking task

**Figure 1:**
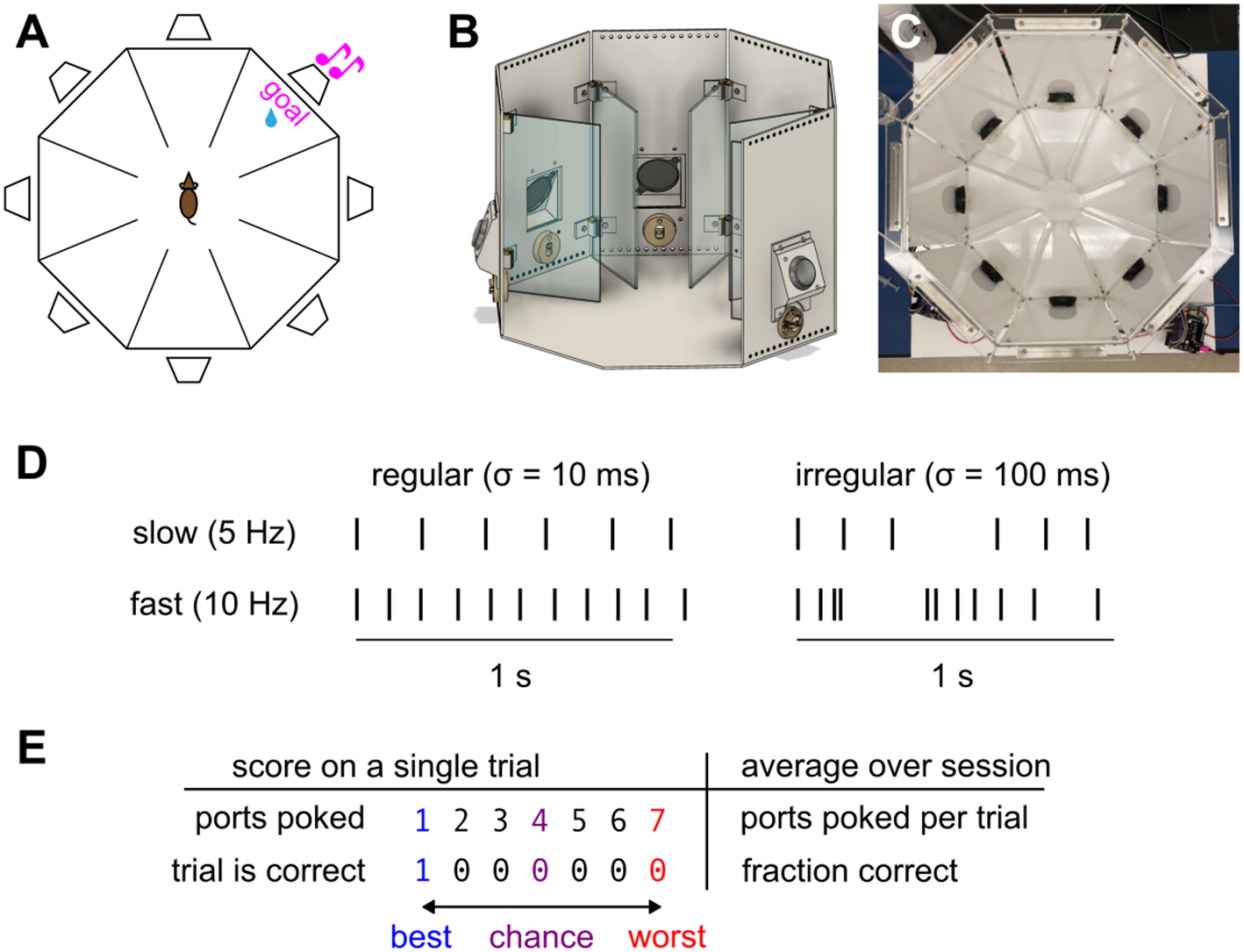
Freely moving mice learn to seek out an ongoing sound stream. (**A**) Multi-chambered octagonal arena for sound-seeking. A speaker (trapezoid) was mounted on each wall above a nosepoke. On each trial, one speaker was randomly selected as the goal (pink). Mice received a water reward for poking the nosepoke below the goal speaker. (**B**) CAD schematic of the arena with two external walls cut away to provide a view from the perspective of the mouse. Speakers are black; nosepokes off-white; and dividers transparent. (**C**) Top-down photograph of the arena. Each side of the octagon was 15 cm and its longest diagonal was 40 cm. Transparent dividers between chambers are nearly invisible in this photo. The entryway into each chamber was 12 cm from the speaker and 5 cm wide. (**D**) The auditory stimulus was an ongoing stream of intermittent noise bursts. The interval between each noise burst was independently drawn from a gamma distribution with mean μ controlling the rate and standard deviation σ controlling the irregularity. (**E**) We scored behavioral performance as the fraction of correct trials and the mean number of ports poked per trial. Pokes into the previously rewarded port were never rewarded and did not affect scoring.

In order to understand how mice coordinate hearing and movement, we began by developing an active auditory task that we named sound-seeking. In this task, mice navigate a multi-chambered arena to track down a sound source. Mice had restricted access to water in order to motivate them to earn water rewards for performing this task^37,38^. During daily behavioral sessions, they were placed in an octagonal arena (Fig. 1A–C) with a speaker mounted on each of its eight sides (*cf.* Ref.^39,40^). A reward port (or “nosepoke”) mounted below each speaker detected “pokes” (intrusions of the mouse’s nose) and could also dispense water in response to pokes that we wished to reward. Transparent plastic walls partitioned the arena into eight peripheral chambers, each with its own speaker and nosepoke, as well as one central chamber from which all others could be reached.

Each session lasted approximately 25 min and comprised a series of trials. On each trial, one of the eight speakers (the “goal”) played a continuous stream of intermittent noise bursts (Fig. 1D). Importantly, the auditory stimulus continued throughout the entire trial until the mouse poked the correct port, in contrast to typical auditory localization tasks that use a single brief stimulus^21,40–44^. The mouse received a reward for poking the port below the goal speaker. Pokes into any other port triggered an error sound (a tritone with a base frequency of 8 kHz). Regardless of incorrect pokes, each trial lasted until the mouse poked the correct port. The mouse did not have to do anything in particular to initiate the next trial, which began 1 s after the previous reward. On each trial the goal speaker was chosen at random. However, the same speaker was never the goal on two consecutive trials because we did not want to reward the mice for perseverating in poking the previously rewarded port.

We quantified how well mice performed this task with two related metrics (Fig. 1E). In calculating both metrics we disregarded pokes into the previously rewarded port, which could never be the goal. First, we calculated the fraction of correct trials, defined as those on which the mouse poked the correct port before any other port. Second, we calculated the mean number of ports poked per trial, ignoring duplicate pokes into the same port. Ports poked per trial varied from 1 on a correct trial to 7 when the mouse poked every other port before the goal (this metric could never reach 8 because we ignored pokes into the previously rewarded port). Chance level performance (e.g., random guessing, poking ports in a fixed order, or any other strategy independent of the sound) would result in four ports poked per trial on average and a fraction correct of 1/7 or 0.143. Perfect performance would result in one port poked per trial (the goal) and a fraction correct of 1. We mainly quantified performance as ports poked per trial because this metric distinguishes between incorrect trials with few versus many incorrect ports poked.

We trained five cohorts of mice under slightly different conditions (Table 1; Supplemental Table 1), not including a pilot cohort of mice used to test the hardware and determine initial task parameters (data not shown). As we optimized the experimental design, we changed between certain cohorts the mouse strain, water regulation protocol used for motivation, acoustic parameters, and/or arena geometry (see Methods), until we arrived at a final and consistent choice of parameters in Cohorts 4 and 5. Despite this variation in parameters, learning was remarkably robust and consistent across animals. In particular, every mouse (n = 69) eventually learned the task well (see next section).

**Table 1:** Mice used in this study.

Cohort	Group	Hearing loss induced	Acoustic parameters	N female	N male	Total N
1	1A	Yes	Fixed	3	2	5
	1B	Yes	Variable	4	6	10
	1C	No	Fixed	2	3	5
2	2A	Yes	Fixed	2	4	6
	2B	No	Fixed	5	5	10
3	3A	No	Fixed	3	2	5
4	4A	Yes	Fixed	2	1	3
	4B	Yes	Variable	2	1	3
	4C	No	Fixed	2	3	5
	4D	No	Variable	2	3	5
5	5A	Yes	Fixed	2	4	6
	5B	Yes	Variable	2	4	6

We trained five cohorts of mice, which were divided into groups (rows of the table). Columns indicate whether each group received hearing loss, whether acoustic parameters were variable vs fixed, and the number of mice. Within each group, mice were always trained in the same way.

To determine which features of the sound affected mice’s decisions, cohorts were divided into groups of mice trained either with variable acoustic parameters (randomly chosen on every trial) or with fixed acoustic parameters (same on every trial). In all cases, the noise bursts were narrowband white noise with 10 ms duration and 3 kHz bandwidth. The intervals between noise bursts were drawn from a gamma distribution, which allowed us to independently vary repetition rate (mean number of noise bursts per second) and irregularity (standard deviation of the interval between noise bursts) (Fig. 1D). We varied irregularity in order to determine whether mice could process sound better when they could precisely predict the time of the next noise burst. For variable acoustic parameter mice in Cohorts 4 and 5, the center frequency was selected from the range 5 to 15 kHz, sound level from 65 to 90 dB SPL (measured 14 cm from the speaker), repetition rate from 3 to 10 Hz, and irregularity from 1 to 100 ms. All of these acoustic parameters were constant within each trial but could vary across trials. For fixed parameter mice in those cohorts, every trial was the same: 4 Hz repetition rate, 31.6 ms irregularity, 70 dB SPL, and 10 kHz center frequency. The speakers were mounted 9.5 cm above the floor and angled at 20 degrees downwards, and therefore mice may have used both binaural horizontal cues and monaural vertical cues induced by pinna. Cohorts 1–3 were trained with slightly different parameters (see Methods).

### Mice reliably learned sound-seeking under a variety of experimental conditions

**Figure 2:**
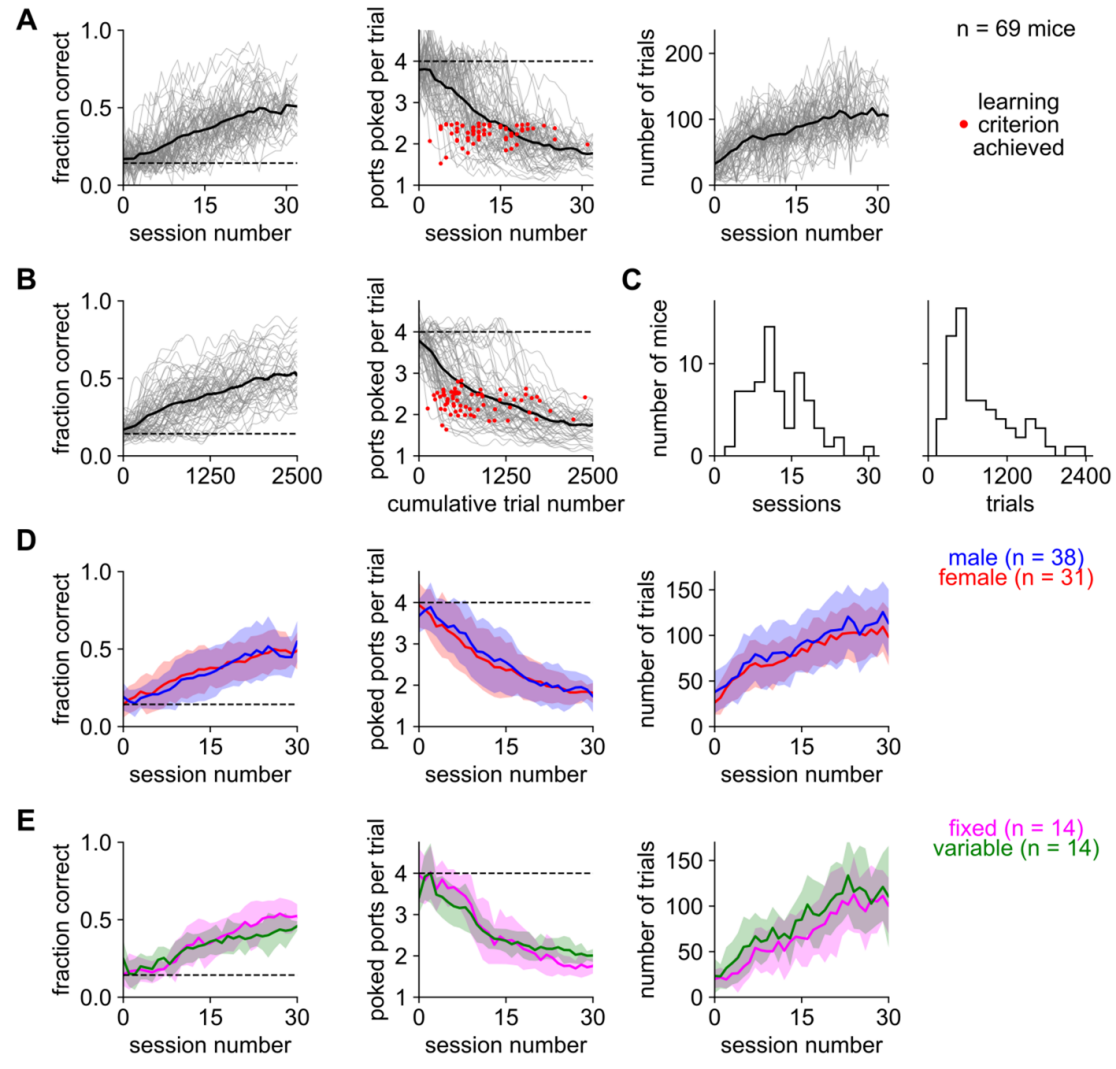
Male and female mice reliably learned either task variant. (**A**) Performance of mice from all cohorts, quantified as fraction correct (left), mean number of ports poked per trial (middle), and number of trials (right) versus the number of training sessions. Gray lines: individual mice. Thick black line: average over mice. Horizontal dashed lines: chance performance. Red dots: first day each mouse achieved the criterion for good performance. (**B**) Same as in (**A**), but now plotted versus the cumulative number of trials over the mouse’s training history. Both performance metrics are smoothed by a Gaussian kernel with a standard deviation of 50 trials. (**C**) Histogram of the time to reach criterion, measured in sessions (left) or trials (right). (**D**) Males (blue) performed more trials than females (red; p < 0.01) but fraction correct and ports poked per trial did not differ (p > 0.05). (**E**) Comparison of mice trained with fixed (magenta) or variable (green) acoustic parameters in cohorts 4 and 5. Mice trained on variable parameters did more trials (p < 0.001) but performed slightly worse on the ports poked per trials metric (p < 0.01); there was no significant difference in the fraction of correct trials (p > 0.05). Statistical significance was assessed as the effect of group in a two-way (group + time) repeated-measures ANOVA. Shaded area: standard deviation.

All mice (n = 69) began at chance but eventually learned the task (Fig. 2A–C). Performance usually plateaued near 0.5 fraction correct (meaning that expert mice poked the correct port first on about half the trials) and near 1.8 ports poked per trial (meaning that they poked an average of 0.8 incorrect ports before the correct one). To divide early learning from late learning, we defined the criterion for good performance as the first session on which each mouse performed better than 2.5 ports poked per trial (red dots in Fig. 2A, B). Mice achieved this criterion in a median of 11 training sessions comprising a total of 597 trials (inter-quartile range: 8–16 sessions and 455–1094 trials; Fig. 2C). The distribution of the required number of trials was more skewed (skewness 1.12) than the distribution of the required number of sessions (skewness 0.67). We speculate that the trial count was skewed by differences in motivation or need for water that were independent of learning rate: some mice performed many more trials but learned in roughly the same number of sessions.

Despite the variation in experimental conditions, including mouse strain and motivational paradigm (deprivation or unpalatability, see “Methods”), performance was roughly similar across Cohorts 1–5 (Supplemental Fig. 1). Because of the multiple differences between cohorts, we cannot conclude whether the strain of the mouse or the motivational paradigm affected learning. However, male and female mice in each group were trained simultaneously and identically, which enabled us to test for any effect of sex (Fig. 2D). Males performed 9.4% more trials than females, likely explained by their larger body size and correspondingly greater need for water. There was no significant difference in the performance of males or females, assessed by either fraction correct or ports poked per trial. In addition, we found only slight differences between groups trained on fixed or variable acoustic parameters (Fig. 2E), suggesting that these two task variants are roughly equal in difficulty. To summarize this section, both male and female mice learned either variant of this task in a few weeks of training.

### Mice were better able to seek out sounds with high repetition rate and low center frequency

**Figure 3:**
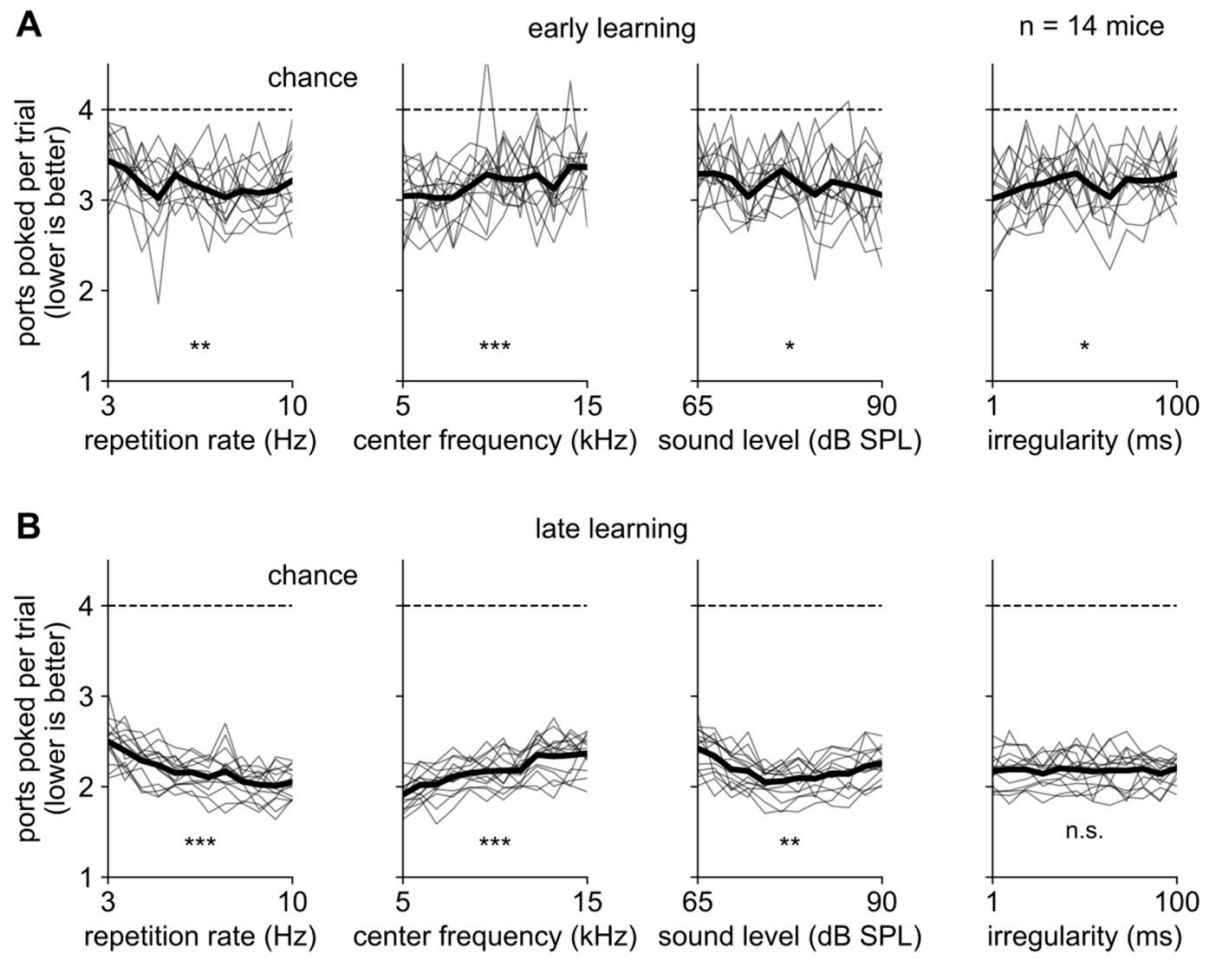
Mice seek out sounds roughly equally well regardless of their acoustic features. (**A**,**B**) Performance versus four acoustic parameters during early learning (**A**) and late learning (**B**). Individual mice: thin gray lines; average: thick black line; chance performance: dashed line. Mice performed significantly better for high repetition rates (first column), low center frequency (second column), and higher sound level (third column) but these effects were small. During early learning, mice were slightly better at irregular sound streams (fourth column). All x-axes are logarithmic. All panels include data from the n = 14 mice in Cohorts 4 and 5 that performed the variable parameters task variant. For each acoustic parameter, statistical significance was assessed with a one-way repeated-measures continuous (not categorical) ANOVA. Throughout this manuscript: * indicates p < 0.05; ** indicates p < 0.01; *** indicates p < 0.001.

In general, mice trained on variable acoustic parameters did similarly well across the range of parameters that we tested (early learning in Fig. 3A; late learning in Fig. 3B). However, they did slightly better on trials with a high repetition rate, indicating they were able to take advantage of the higher information rate on these trials. They also did slightly better on louder sounds, though in late learning performance appeared to plateau or even worsen at the highest sound levels (Fig. 3B, third panel). In early learning, mice did slightly better at more irregular sound streams (Fig. 3A, fourth panel); we speculate that irregular sound streams may have been more salient, but in any case this effect was small and disappeared by late learning.

Perhaps surprisingly, mice were better at seeking out low-frequency sounds (Fig. 3, second column). While this effect was small, it was unexpected because mice localizing sound rely mostly on interaural level differences, which are more prominent at high frequencies^45^, and because a previous study in mice reported worse spatial acuity at low frequencies^46^. However, another study found relatively good performance at low frequencies, likely due to interaural level differences^47^. (The mouse head is too small to exploit interaural timing differences, the other main binaural cue. Monaural spectral cues are mainly important for high frequencies and for localization in elevation rather than azimuth^47^.) In our task, we speculate that the intervening divider walls may more effectively restrict low-frequency sounds to a single chamber (the goal) or change the reverberation pattern. While reverberation is generally thought to impede localization, in some cases it can help^48^.

Notwithstanding these relatively small differences, mice performed roughly equally well for all sounds. In other words, within the range of parameters that we tested, task performance was mainly limited by errors that were unrelated to the acoustic properties of the sound.

### Resiliency to unilateral but not bilateral hearing loss

**Figure 4:**
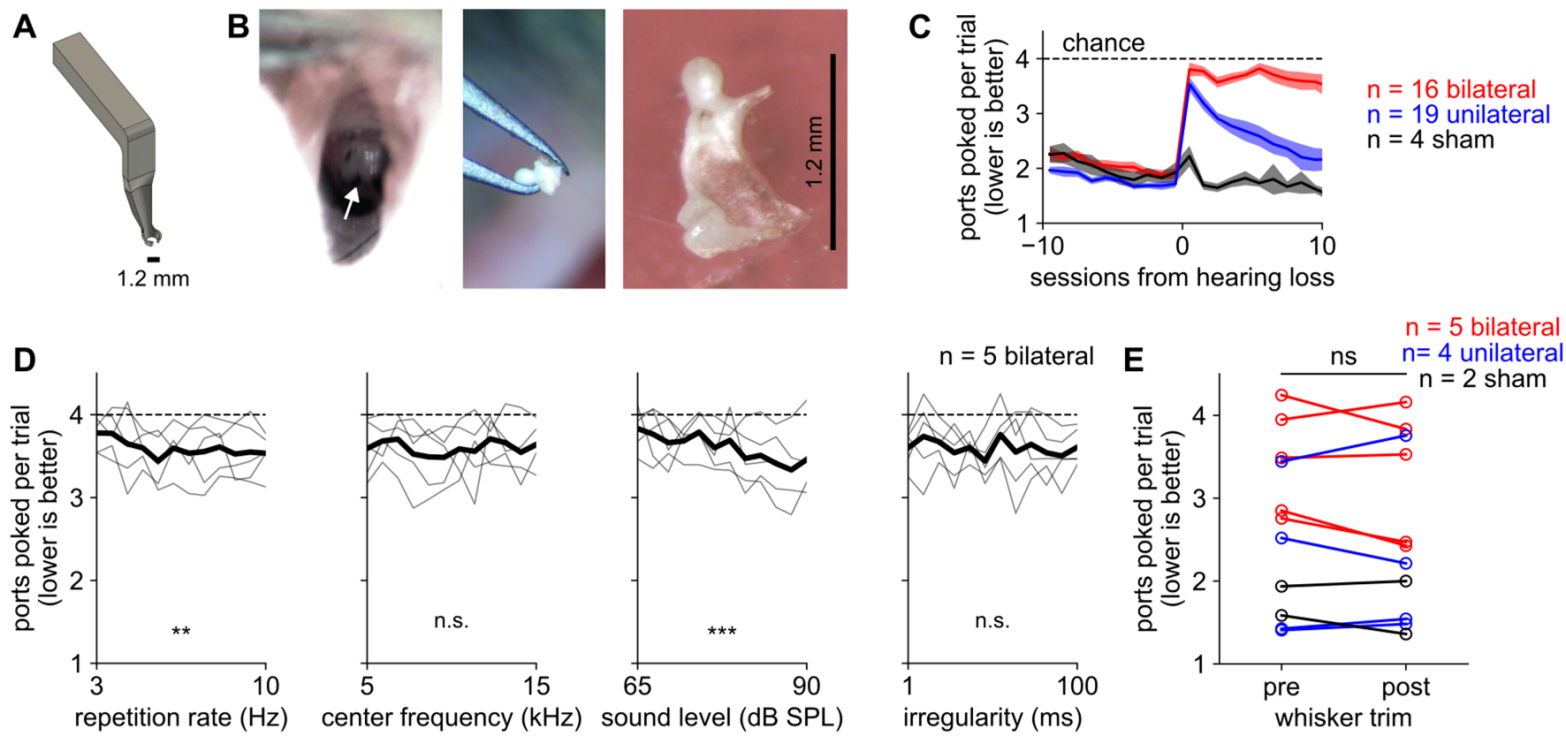
Sound-seeking recovers after unilateral but not bilateral hearing loss. (**A**) Schematic of a 3d-printed miniature ear speculum for mice that we designed to visualize the middle ear. (**B**) View into the ear canal showing malleus in situ (white arrow, left), removal (middle), and higher magnification view of removed malleus (right). The manubrium has broken off this example malleus. (**C**) Ports poked per trial increased (i.e., performance worsened) after hearing loss (p < 0.001 for bilateral and unilateral; p > 0.05 for sham). Bilateral mice performed worse than both other groups (p < 0.001) and unilateral performed worse than sham (p < 0.001). Unilateral mice improved over time after hearing loss (p < 0.001) but bilateral and sham mice did not (p > 0.05). We used ANOVA to assess significance between groups and during recovery and a post-hoc paired t-test before and after hearing loss for each group. This panel includes n = 39 mice from Cohorts 1–5 who performed either the fixed or variable parameters task and received hearing loss surgery (Table 1). (**D**) After bilateral hearing loss, performance is overall poor but it is slightly better for louder sounds (third column) and higher repetition rate (first column). Conventions as in Fig. 3. This panel includes n = 5 bilateral mice from Cohorts 4 and 5 who performed the variable parameters task. (**E**) Performance did not change in the subset of mice who received a whisker trim (p > 0.05; paired t-test).

We next asked how mice were affected by hearing loss. Unilateral or bilateral conductive hearing loss can arise from childhood ear infections, among other reasons^49,50^. After mice had learned the sound-seeking task, we induced hearing loss in one or both ears by surgical extirpation (i.e., removal) of the malleus (Fig. 4A, B). Without the malleus, animals are not completely deaf but the intensity threshold for detecting sound is permanently elevated by 30–55 dB^51,52^ across all frequencies. This surgery is a model of conductive hearing loss because the malleus and the rest of the ossicular chain conduct acoustic vibrations to the cochlea^51–55^.

We randomly selected mice for left, right, bilateral, or sham surgery. For bilateral surgery and unilateral surgery on the deprived side, the tympanic membrane was broken and the malleus removed. For sham surgery and unilateral surgery on the spared side, the tympanic membrane and malleus were only visualized and not disturbed. In all cases, we used the same anesthesia, analgesia, and surgical scrub of the ear canal. We confirmed that unilateral and bilateral malleus removal affected hearing by measuring the auditory brainstem response^56–58^ (ABR; Supplemental Fig. 2).

After hearing loss, we tested mice on the sound-seeking task that they had previously learned. Mice with sham hearing loss showed either no change in performance or a minor impairment on only the first session after surgery (black line; Fig. 4C). Mice with bilateral hearing loss were profoundly impaired to near chance levels without significant improvement for up to 10 sessions after the surgery (red line; Fig. 4C). Strikingly, mice with unilateral hearing loss demonstrated an early and substantial impairment, but over 5–10 post-operative behavioral sessions their performance almost fully recovered (blue line; Fig. 4C). These results were similar whether returns to the previously rewarded port were included or excluded (Supplemental Fig. 3). Thus, mice rapidly regain their sound-seeking ability after losing auditory input on one side, but not if both sides are deprived.

Because malleus removal should raise the threshold for detecting sound without completely deafening the mouse, we considered the possibility that bilateral mice would retain an ability to find loud sounds but not soft sounds. We tested this hypothesis in the subset of mice in Groups 4B and 5B (Table 1) that were trained on the variable parameters version of the task, in which sound level and other acoustic parameters were varied from trial to trial. Groups 4B and 5B included n = 5 bilateral mice but too few unilateral (n = 2) and sham (n = 2) mice to analyze in this way. The bilateral mice showed a small but significant increase in performance on trials with louder sounds (Fig. 4D, third column). It is possible that amplifying the sound further to compensate for the increased auditory thresholds after malleus removal (e.g., by 30–55 dB) might have restored performance, but we did not test this.

We also considered whether unilateral mice might have recovered their sound-seeking ability by learning to detect sound waves with their whiskers instead^59,60^. To test this possibility, after at least 13 post-surgical days had passed we cut off all of the macrovibrissae on a subset of mice. Their performance was unaffected by trimming whiskers, showing that they did not use whisker input to perform this task (Fig. 4E).

Finally, we considered the possibility that mice exploited other tactile cues, such as feeling floor vibrations. Any such non-auditory cues would remain available after bilateral malleus removal, and yet those bilateral mice performed nearly at chance (Fig. 4C). We cannot exclude the possibility that the small amount of above-chance performance in bilateral mice arose from tactile input, but it could also have arisen from the remaining auditory input. In any case, the robust impact on performance of depriving auditory input means that non-auditory contributions to sound-seeking are slight at most.

The most likely explanation for the recovery in unilateral but not bilateral mice is that unilateral mice learned to make better use of their remaining auditory input, a strategy unavailable to the bilateral mice. For instance, unilateral mice might have learned to reply on spared monaural cues from the healthy ear (such as spectral differences) or to recalibrate their interpretation of binaural cues after unilateral threshold changes (cf. reweighting and remapping^61^). Narrower bandwidth stimuli could be used to probe the role of spectral cues in future experiments.

### The acoustic startle response does not recover after hearing loss

**Figure 5:**
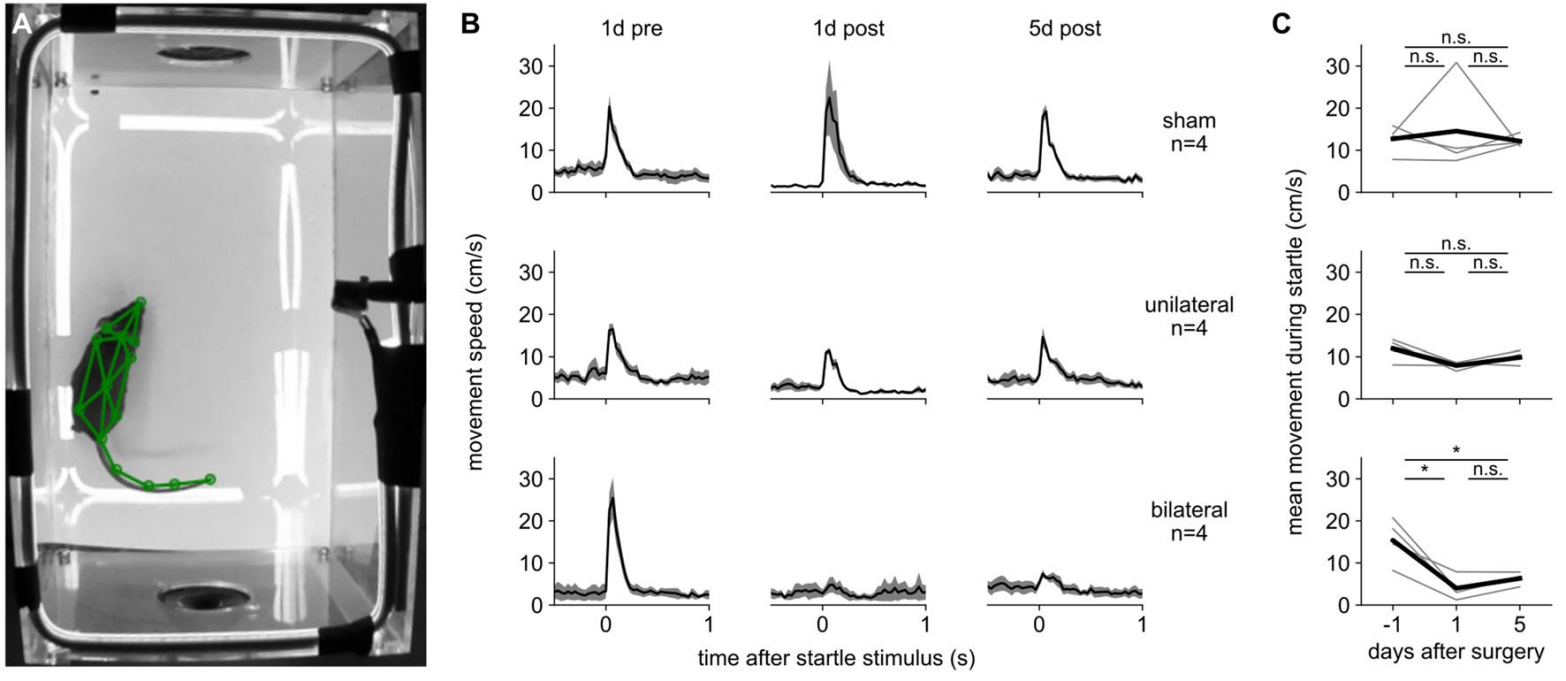
The auditory startle response is robust to unilateral hearing loss and does not recover after bilateral hearing loss. (**A**) Example video frame showing startle test arena. Speakers are visible at both ends of arena. Green lines: tracked body parts. (**B**) Acoustic startle response, quantified as full-body movement versus time from the onset of the startle-eliciting stimulus, at 1 day before, 1 day after, and 5 days after surgery (columns) for sham, unilateral, and bilateral mice (rows). Shaded area: SEM over individual mice. (**C**) Acoustic startle response, quantified as the mean movement speed over the first five frames (167 ms) after the startle-eliciting stimulus. The response did not change significantly after sham or unilateral hearing loss (top and middle), but it was significantly reduced after bilateral hearing loss (lower panel; p < 0.05, paired t-test). Thin lines: individual mice. Thick line: mean over mice.

We asked whether recovery from hearing loss was specific to sound-seeking or would also occur in other auditory behaviors, perhaps reflecting a generalized recovery of hearing ability over time. Indeed, central auditory pathways in the inferior colliculus and auditory cortex can robustly restore sensitivity even in the face of intensive sensory deprivation^62,63^. To assess whether mice could respond to sound after hearing loss in a distinct auditory behavior, we assessed the startle response, which is a brief whole-body muscular contraction in response to an unexpected sound at relatively high level^64^. To measure startle, we tracked body movements in video using the pose-tracking algorithm SLEAP^65^ (Fig. 5A). Startle tests occurred in a dedicated behavioral chamber, not in the sound-seeking arena, with a background of continuous white noise at 60 dB SPL. The startle-eliciting stimulus was a 100 ms white noise burst with a level of 80 or 90 dB SPL. Because these two levels elicited similar results we combined them in our analysis.

We measured the startle response in Cohort 5 on three days, one pre-operative to establish baseline and two post-operative to assess the effects of hearing loss (Fig. 5B, C), over the same period of time that we tested the same mice on sound-seeking. After sham hearing loss, the startle response was generally unchanged at days 1 and 5 (although one outlier mouse was greatly enhanced at day 1 only), demonstrating that startle does not habituate or diminish over time under these experimental conditions. After bilateral hearing loss, the startle response was nearly abolished at post-operative days 1 and 5. After unilateral hearing loss, the startle response was nearly unaffected at days 1 and 5.

Because unilateral mice did not lose their startle response, we cannot assess their ability to recover it, but we can conclude that not all auditory behaviors are sensitive to unilateral hearing loss. While it is possible that additional testing of unilateral mice would have revealed a subtle impairment and/or recovery of the startle response, qualitatively this behavior was much less affected by unilateral hearing loss than sound-seeking was. We speculate that the startle response may rely on circuits that require only input from a single ear, whereas recovery of sound-seeking may require time to recalibrate central auditory or motor circuits.

Because bilateral malleus removal is not a complete deafening, it is likely that a louder startle stimulus could still have elicited a startle in bilateral mice. We used a fairly low sound level (cf. Ref.^66^) because it was the lowest level that was still capable of eliciting a robust startle response in a pilot cohort of healthy animals (data not shown). Nonetheless, because bilateral mice lost their startle response and did not recover it, we find no evidence for generalized recovery of hearing over time (e.g., through residual sensitivity of the cochlea and gain control in auditory afferents). Thus, the strong recovery in sound-seeking after unilateral hearing loss is unlikely to be explained by a generalized recovery of hearing ability.

### After bilateral hearing loss, mice took a faster but less straight path to the goal

Because the peripheral auditory system cannot recover full auditory sensitivity after malleus removal, we next asked what behavioral changes might explain how mice recovered their sound-seeking ability. For instance, mice might have taken a different path through the arena, or sampled each chamber in different ways. To track the paths mice took, we again used the pose-tracking software SLEAP to track body position during sound-seeking in 18 mice (7 bilateral, 7 unilateral, and 4 sham from Cohorts 4 and 5) from initial learning through expert performance and recovery from hearing loss.

**Figure 6:**
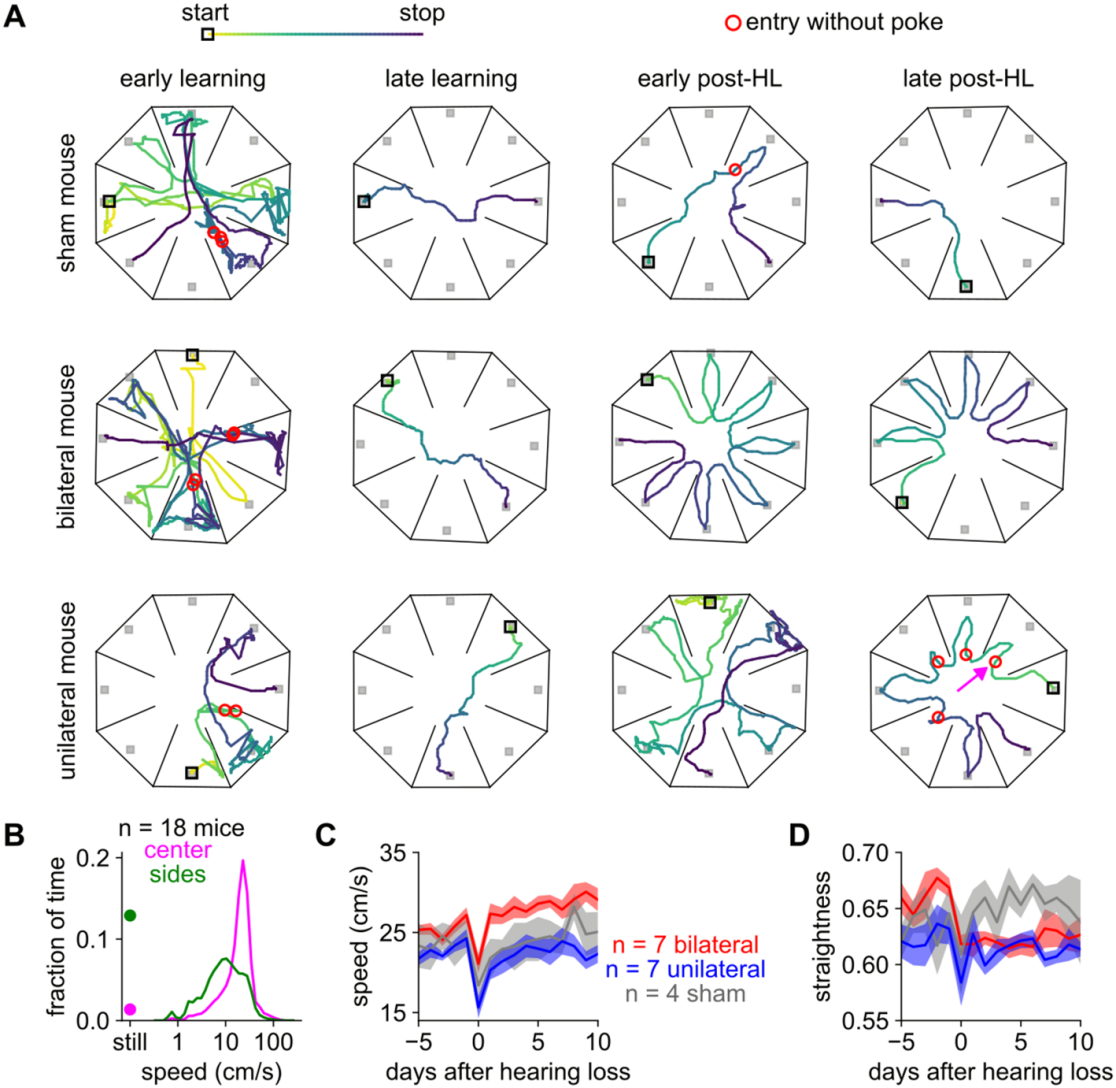
After bilateral hearing loss, mice move more quickly but in less straight paths through the center. (**A**) Example trials from different mice (rows) taken at four different timepoints over behavioral testing (columns). Colored line: mouse trajectory from early in trial (warm colors) to late (cool colors). Open black square: start of path. Filled gray squares: port locations. Red circles: entries without poke (checks). Pink arrow in lower right indicates a particular check illustrated in Fig. 7A. (**B**) Histogram of the speed of n = 18 mice on each frame while in the center (pink) or in the side chambers (green). In the side chambers mice were still for 12.9% of the time whereas in the center they were still for only 1.4% of the time (filled circles). (**C**) Speed through the center chamber. After hearing loss, only bilateral mice (red) increased their speed (p < 0.05). Compared to sham, bilateral mice were faster (p < 0.001) and unilateral were slower (p < 0.01). (**D**) Straightness of paths (defined in text) through the center chamber. After hearing loss, paths of bilateral mice decreased in straightness (p < 0.05) and were less straight than those of sham mice (p < 0.001). The paths of unilateral and sham mice did not significantly change after hearing loss and the paths of unilateral mice did not significantly differ from either of the other groups (p > 0.05). In (**C,D**), we used ANOVA to assess significance between groups and during recovery and a post-hoc paired t-test before and after hearing loss for each group.

Qualitatively, mice followed an increasingly straight path to the goal as they learned the task, but a less straight path after hearing loss. Early in learning, mice generally navigated the arena with a tortuous and self-crossing path, entering the same chambers and nosepokes multiple times (example trials in first column of Fig. 6A). Later in learning, mice were less likely to poke unrewarded nosepokes, less likely to enter the same chamber multiple times, and generally took a more direct path to the goal (second column of Fig. 6A). After hearing loss, mice took a less straight path to the goal (last two columns of Fig. 6A): both unilateral and bilateral mice often cycled in one direction around the arena to visit each chamber in turn, although unilateral mice appeared to be more likely to exit non-goal chambers without poking (see next section).

Mice occasionally spent long periods of time disengaged from the task in the peripheral chambers (consuming reward, grooming, etc.) but they generally moved through the center without stopping (Fig. 6B). Therefore, we focused our analysis on the speed and straightness of paths taken through the center. We defined the straightness of each path as the length of the straight line from the path’s start to its end (D_straight_) divided by the length of the actual path taken (D_actual_). By this metric, all paths have straightness between zero (a looping path that returns to its own starting point) and one (a straight line).

After hearing loss, bilateral mice moved more quickly through the center than either of the other groups and their paths were less straight than those of sham mice (red lines in Fig. 6C, D). One possible interpretation is that bilateral mice were able to move more quickly because they executed a routinized and cyclical search of the arena that did not require listening or making choices about where to go next, whereas sham and unilateral mice moved more slowly because they continued to rely on auditory input to guide their movement. This possibility led us to next ask what sort of behavior might reflect the collection and use of auditory input to regulate movement.

### Mice with unilateral hearing loss increasingly relied on checking each chamber for sound

We noted that mice would briefly enter a chamber without poking the port (red circles in Fig. 6A), a behavior we called “checking” because mice appeared to enter the chamber in order to check whether that speaker was playing sound. Although checking also occurred before hearing loss, we hypothesized that mice increasingly relied on checking as a compensatory strategy for unilateral hearing loss.

**Figure 7:**
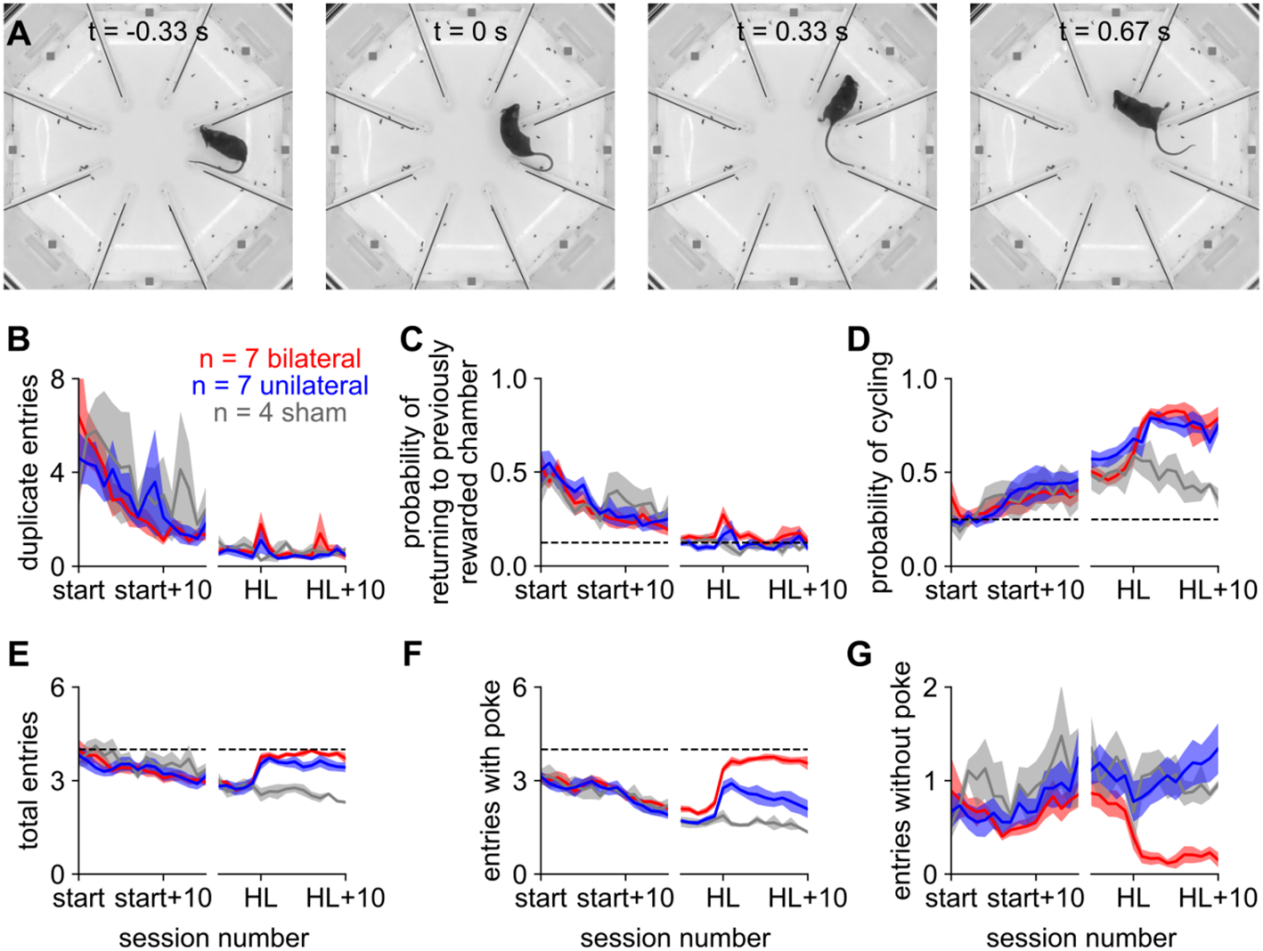
After unilateral hearing loss, entries without poke (“checks”) increase. (**A**) Example frames from 0.33 s before to 0.67 s after the check indicated by the pink arrow in the lower right of Fig. 6A. The mouse briefly enters the northeast chamber but leaves without poking. (**B**) In (**B–G**), the x-axis is broken into two epochs: a learning epoch comprising the first 15 sessions of training on the left, and a peri-hearing loss epoch comprising the 5 sessions before and 10 sessions after hearing loss surgery on the right. Duplicate chamber entries per trial decreased over learning (p < 0.001). After hearing loss, this metric did not significantly change for any group (p > 0.05). (**C**) Fraction of trials on which the mouse returned to the previously rewarded chamber. This metric significantly decreased over learning (p < 0.001). After hearing loss, it did not significantly increase for any group (p > 0.05). (**D**) Probability of “cycling” (i.e., entering a chamber adjacent to the one just exited). Cycling significantly increased over learning (p < 0.001). After hearing loss, it was significantly more common in bilateral and unilateral mice than in sham (p < 0.001). (**E**) Mean number of chamber entries per trial, excluding duplicate entries and returns to the previously rewarded chamber. After hearing loss, this metric was significantly higher for bilateral and unilateral mice than for sham (p < 0.001), and in both of those groups it approached the chance level of 4 (dashed line). All chamber entries in (**E**) are subdivided into two categories in (**F,G**). (**F**) Mean number of chamber entries with poke. This metric is essentially the same as plotting the number of ports poked (Fig. 4C). These entries increased after unilateral (p < 0.01) and bilateral (p < 0.001) but not sham hearing loss. Over the recovery period after hearing loss, these entries decreased for unilateral (p < 0.001) but not bilateral mice. (**G**) Mean number of chamber entries without poke (i.e., checks). These entries increased over learning (p < 0.01). They decreased after bilateral (p < 0.01) but not unilateral or sham hearing loss. During the recovery period after hearing loss, these entries increased over time for unilateral mice (p < 0.001). They were less frequent in bilateral mice than in either of the other two groups (p < 0.001). In (**B–G**), we used ANOVA to assess significance between groups, during learning, and during recovery, and a post-hoc paired t-test before and after hearing loss for each group.

To quantify checking, we first calculated for each trial the sequence of “chamber entries”, defined as the first video frame on which the snout passed into a chamber (example: Fig. 7A). Duplicate entries (i.e., re-entering a chamber that had already been visited on the same trial) declined over learning and stayed stable after hearing loss (Fig. 7B), as did entries into the chamber containing the previously rewarded port (Fig. 7C). Cycling entries (*i.e.*, entering a chamber directly adjacent to the one just exited) significantly increased over learning; after hearing loss, cycling was more common in bilateral and unilateral compared to sham (Fig. 7D). Decreased path straightness likely reflected this increase in cycling. Thus, we find notable differences in the order in which chambers were entered: naive mice enter chambers unsystematically and repeatedly; expert mice cycle and avoid previously visited chambers; mice with hearing loss cycle even more. Cycling may be an adaptive strategy because cycling guarantees the mouse will find the goal before it returns to a chamber previously visited on that trial (e.g., bilateral and unilateral trajectories in rightmost column of Fig. 6A).

Finally, we quantified our observation that mice often “checked” chambers by briefly entering the chamber but leaving without poking the port. We divided chamber entries into two categories: those that eventually culminated in a nosepoke (“entry with poke”) and those that did not (“entry without poke”, i.e. a check). Excluding duplicate entries and entries into the previously rewarded port, the total number of chambers entered per trial began at around 4 (commensurate with entering chambers at random), decreased over learning, and increased to the chance level again after either form of hearing loss (Fig. 7E). After bilateral hearing loss, entries with poke increased and entries without poke (checks) almost entirely ceased (Fig. 7F, G, red line). In contrast, after unilateral hearing loss entries without poke increased (Fig. 7G, blue line). That is, while both bilateral and unilateral mice visit unrewarded chambers at chance levels (Fig. 7E), unilateral mice are better able to refrain from poking after entering an incorrect chamber (Fig. 7G).

One possible interpretation is that bilateral mice lost the ability to find the goal by any means other than an exhaustive search, poking every port in turn. In contrast, while unilateral mice did cease to navigate directly to the sound (an ability that may rely on binaural cues), they were still able to check each chamber for sound without actually poking the port. Thus, in the absence of the binaural cues that would normally enable mice to determine the location of sound, they may instead rely on a movement-based strategy of checking each possible location for sound^67^.

## Discussion

In this study, we developed a sound-seeking task in which mice sought out a continuous sound source through ongoing movement of the head and body. Over training, mice learned to take a more efficient path to the goal that visited fewer chambers. After bilateral hearing loss, this path became routinized as mice generally visited each port in turn, leading to chance level performance. In contrast, mice with unilateral hearing loss were transiently impaired but recovered their performance over time. We did not observe the same pattern of transient impairment followed by recovery in an innate behavior, the auditory startle response. We speculate that unilateral mice, no longer able to exploit binaural cues to navigate directly to the goal port, instead checked each chamber and relied on monaural input to release a nosepoke motor response. Overall, this work provides a platform to study how the brain can exploit movement strategies to build resilience to sensory impairment, with implications for rehabilitation and recovery in auditory and other modalities.

### Active sound-seeking versus passive sound localization

Sound-seeking challenges mice to integrate auditory input with movement of the head and body in order to find an ongoing sound source. Our results were presaged by early work in which mice were better able to localize ongoing sound if they could move toward it^39^. Sound-seeking sharply contrasts with what we call “passive sound localization” tasks, which are designed to separate body movement from hearing. For instance, passive studies might physically restrain the subject^42,47,68^, train them to hold still during the sound^44,69,70^, or use sounds so brief that they terminate before the subject can move^40,41,71–73^. For example, Ref.^71^ writes “*Brief stimuli *[*were used*]* to eliminate the possibility of scanning movements of the head or body as a localization strategy.*” These studies removed motor feedback in order to expose the precise sensory cues that animals can use to compute sound location^74^, as well as the underlying brainstem and cortical neural circuitry^45,75–79^. Our approach differs in three important ways.

First, rather than probe perception when movement is impossible, sound-seeking models what free animals might naturally do, such as during pup retrieval^34,35^. Fundamentally, accurate sound localization requires information from at least two points in space. This information can be obtained with simultaneous input from multiple sensors (e.g., the two ears) or alternatively from a single sensor moving through space over time; the latter is only possible if the subject is free to move and the stimulus is ongoing^16^. For instance, the subject could move the head to place the sound in a region of the spatial field where acuity is highest^47^. Rather than enframing the process of decision-making as a feed-forward sequence of sensory processing, deliberation, and finally action selection, we favor a recurrent model in which perception and action guide each other^31,80–83^.

Second, most studies of sound localization take place in anechoic (echo-suppressing) chambers with minimal physical structure that could impede the passage of sound waves^77^. Such a design is better for measuring the exact acoustic features that enter the auditory system, but ultimately it is not representative of the natural environment. Humans and animals live in reverberant, multi-chambered environments such as buildings and burrows^48,84^ and interpreting the resulting echoes is an important function of auditory neural circuitry^45^. Our arena was built with acrylic plastic, which reflects sound. The presence of echoes can either increase or decrease task difficulty^48^. For instance, sound-seeking mice may exploit the physical structure of the arena by ducking briefly into each chamber in order to check the sound level, as opposed to relying on binaural cues in the center of the arena. Exploiting the physical structure of the environment to simplify neural computations is an adaptive strategy for cognition^85,86^.

Third, in our task mice registered their decision by physically approaching the sound, rather than by licking or pressing a lever. While the nosepoke itself is of course artificial, natural behavior often involves approaching a sound (as in pup retrieval) or fleeing a sound (as in escape from predators). When perception and action are viewed as separate processes, the mode of response would seem to be irrelevant to sensory processing. However, from the perspective that the brain’s function is to produce adaptive action, the mode of response may drastically alter the underlying neural computations. For instance, classic work in bushbaby suggested that auditory cortex was required specifically to link auditory perception with an approach response^87^, as opposed to auditory localization per se (although see Ref.^71^). Directing the eyes toward a sound enhances localization^88^, and it has even been suggested that auditory localization evolved to support directing the eyes toward important objects^89,90^. Sound-seeking unifies the sensory problem of localizing sound with the motor problem of orienting and moving toward it.

### Spatial strategies for foraging for rewards

Throughout learning and especially after hearing loss, mice increasingly cycled through the chambers in a clockwise or counterclockwise spatial sequence. This strategy is reminiscent of traplining, a form of foraging in which animals revisit potential sites for reward in a regular spatial order^91^. According to the way we measured performance, cycling is a chance-level strategy, no more effective than randomly choosing ports. But from the animal’s perspective, cycling may be more efficient or cognitively easier because the same turning movement is employed over and over, and because the memory of the ports previously checked is embodied in the trajectory taken. In addition, mice may innately prefer the cycling strategy because it hugs the walls and avoids entering the open center of the arena^92^; thus, sound-seeking may require mice to balance phonotaxis with thigmotaxis. A similar sound-seeking paradigm using mouse pups as the reward has been developed as a model of parental pup retrieval. That work demonstrated the gradual replacement of a spatial strategy with an auditory-guided strategy, likely guided by the projection from prefrontal to auditory cortex^93^.

While perseverative spatial biases limit performance in laboratory tasks with randomized reward locations, they are likely adaptive under natural conditions where rewards are rarely random and often reoccur. While on the one hand parietal cortex encodes trial history and causally drives perseverative responses^94^, it is also possible that other brain regions encode trial history for the purpose of overcoming these biases and producing the unbiased responses necessary for success in laboratory tasks. For instance, primary sensory cortex can encode choice and reward history over trials^8^, and the prefrontal cortex both encodes auditory responses and is necessary for task switching^95–97^. Sound-seeking provides an experimental platform for understanding how neural circuits update, balance, and select distinct strategies throughout learning and in response to sensory loss.

### Recovery from hearing loss

Hearing loss lowers the signal-to-noise ratio (SNR) at the input to the auditory system. Adaptive filtering in neural circuits can extract the most signal out of this weakened input^98^. For instance, central auditory pathways can amplify the remaining input and restore some behavioral abilities, one possible explanation for the recovery we observe. However, the data processing inequality means that no amount of neural processing can restore hearing in full; informally, “turning up the volume” can only do so much because it amplifies noise as well as signal^62,63,99^. The same logic limits the theoretical efficacy of hearing aids. Moreover, the cognitive load associated with difficult auditory processing might worsen or accelerate dementia^100,101^.

However, body movement is a potent way to enhance auditory processing that could help the healthy and hearing-impaired alike. For instance, in very noisy environments one might turn an ear toward the speaker in order to better hear their voice; this response can be either innate or learned, and it can benefit both healthy and hearing-impaired listeners^23^. To find out which smoke alarm is chirping, one is more likely to explore the house room by room than to sit stationary and compute interaural localization cues^67^. Finally, interaural time and localization cues are identical for a sound exactly in front and exactly behind the listener, but this ambiguity can be readily resolved by moving the head to a new angle, which will generate very different cues for the two previously ambiguous cases^17,18,26^. These movement strategies improve SNR by moving the sensor rather than by implementing complex algorithms to process noisy data.

Movement is a natural, innate strategy for improving our hearing. Translationally, a better understanding of these movement strategies and how they can be taught through physical therapy could provide a less invasive, less expensive, and faster treatment plan. Scientifically, this leads to the counter-intuitive idea that recovering from sensory disorder may rely on plasticity in motor circuits. The sound-seeking task provides a platform to study how networks of sensory and motor brain areas work together in order to enable the ongoing interaction between perception and action that we call behavior.

## Methods

All animal experiments were conducted under the guidance and approval of the Emory University Institutional Animal Care and Use Committee under protocol number 202100154 in compliance with the ARRIVE guidelines.

### Behavioral training

Mice were purchased from Jackson Laboratories or bred in a pathogen-free barrier facility (additional details given in Supplemental Table 1). All mice used in this study were CBA/CaJ (Jackson Labs 000654), an F1 hybrid of C57BL/6J (000664) and CBA/CaJ, or an F1 hybrid of C57BL/6J and CBA/J (000656). They were kept in single-sex cages after weaning. We generally provided enrichment such as running wheels and manzanita sticks, and we usually avoided selecting mice with signs of barbering or who were resistant to handling. We always attempted to use males and females in equal proportion, though this was not always possible given the vagaries of mouse breeding.

Training began with 2–5 days of handling, in which the experimenter habituated the mouse to being picked up and weighed. Then we began water regulation, which could be of one of two types as specified in Table 1. For water restriction, the water bottle was removed from the cage, and mice only received water during their daily behavioral training. We monitored how much water they received during the experiment, and provided a calibrated amount of water as needed to ensure that they always received their daily water ration of at least 4% of their body weight per day. We used a system of weight thresholds and daily health checks to ensure that mice remained healthy on this regimen, and in some cases increased their daily ration as needed to maintain a healthy weight and disposition. As an alternative to water restriction, we also used regulation by unpalatability, in which case mice had unlimited access to water containing 1.5–3% citric acid (Sigma) in the standard home cage bottle. Citric acid is a food preservative that lends water a sour and unpalatable flavor without affecting the subject's health, even over long-term consumption^37,38^. These mice were motivated to perform the same task for the same water rewards because the pure water tasted better than their home cage water. Typically, mice began at 1.5% citric acid and were increased to higher concentrations if their trial count was substantially lower than usual. We lowered the water reward size as mice became better at the task to encourage them to perform more trials.

After 1–2 days on this water regimen, mice began “pre-training” which lasted about 3–7 days (one session per day) and taught them to use the nosepokes. The first stage of pre-training was “manual poke training” in which they were introduced to the behavioral arena. In this stage we placed ~ 0.1 mL of water inside the nosepokes using a syringe so that they would learn to associate the nosepokes with reward. After mice learned to reliably obtain their daily ration from the nosepokes, they began “automated poke training”. In this stage, no sounds were ever played, and every poke into a nosepoke triggered the release of a small water reward (~ 5 to 15 μL), with the exception that the same nosepoke never delivered a reward twice in a row. Mice quickly learned to visit nosepokes serially, often in a cycling pattern. Once they learned to reliably obtain their daily ration in this way (typically 2—5 days), pre-training ended and they began training on the sound-seeking task described in the main text. At this point in their training, the median mouse was 91 days old (IQR: 83–115).

The behavioral system was controlled with our fork (https://github.com/Rodgers-PAC-Lab/autopilot) of the Autopilot behavioral control system^102^. Briefly, the experimenter used a graphical user interface on a desktop PC that communicated with a Raspberry Pi microcontroller over a wireless network. This “parent” Raspberry Pi chose the stimulus parameters on each trial and communicated them to four Raspberry Pi “children”. Each child Pi controlled two speakers (either Multicomp Pro MCPCT-G5100-4139 or Almencla Ultrasonic Tweeter 2-inch Waterproof Piezo Horn) using a HiFiBerry Amp2 audio card and two Autopilot nosepokes using custom-built printed circuit boards. The child Pi communicated to the parent Pi every nosepoke and reward, and the parent Pi reported these values to the graphical user interface on the desktop PC, where they were stored in an HDF5 file for later analysis. On rare occasions data from individual trials or individual sessions was lost due to corruption in the HDF5 file, which may contribute to a slight underestimate of the training time required in the earlier cohorts.

We used an ultrasonic microphone to measure the sound power at every frequency, and we corrected the amplitude of the sound for the frequency response of the sound card and speaker to ensure that the sound pressure level was consistent across frequencies. Some earlier cohorts were trained without this calibration procedure, but these speakers had an frequency response that was already fairly flat within the range tested here. The arena was constructed of acrylic and custom 3d-printed parts. The behavioral training was conducted inside a custom-designed sound-attenuating chamber (Item24). Water rewards were delivered to the nosepoke by opening a solenoid valve (The Lee Co LHDA05311115H).

### Speaker calibration

We calibrated the end-to-end frequency response of our audio system using an industrial microphone (Hottinger Brüel & Kjær 2829 and 4939). The tip of the microphone was placed 14 cm from the speaker, 10 cm above the floor, parallel to the floor, and pointing directly at the wall containing the speaker. In this location, the microphone was right at the entrance to the peripheral chamber. During calibration we recorded full-bandwidth white noise, measured the spectrum of the received acoustic power, and calculated the correction factor needed at each frequency to reach a uniform spectrum. During the behavioral session, we applied this correction factor to the sounds in software before sending them through the audio card. After this procedure, the frequency response of our audio system was within ± 10 dB over the range 5 to 80 kHz, and within ± 5 dB over the range used for behavioral testing (5–20 kHz).

The sound level of the click stimulus used for the auditory brainstem response (ABR) was measured using a different microphone (BAFX 3370) that applied A-weighting rather than a flat frequency response. Therefore we report this sound level in units of dBA instead of dB SPL.

### Hearing loss surgery

To induce hearing loss, we surgically removed the malleus on one or both sides of the head. Briefly, animals were administered pre-operative extended-release buprenorphine and either carprofen or extended-release meloxicam. They were anesthetized with isoflurane throughout the procedure. We used aseptic technique and ensured sufficient surgical depth of anesthesia to abolish the toe pinch reflex. The hair around the ear was shaved or removed with depilatory cream. The skin of the ear canal was disinfected with three alternating washes of Betadine and 70% ethanol. Under a surgical microscope, we visualized the tympanic membrane using fine forceps and/or a custom-designed 3D-printed miniature ear speculum. The tympanic membrane was broken and the malleus removed with fine forceps, but the other ossicles were left in place. Finally the ear canal was flushed with sterile saline. For sham surgeries, the procedure was identical up to and including visualization of the ossicles and flushing with saline, but the tympanic membrane and all ossicles were left intact and in place. The experimenters training the mice were generally blinded to the form of hearing loss experienced by each mouse. The first post-surgical session occurred 1–3 days after the surgery. We did not observe any difference within this time range, although this study was not designed to detect such an effect.

### Auditory brainstem response (ABR) measurement

We measured the physiological function of the inner ear and auditory nerve by measuring the ABR^56^. Mice were anesthetized with isoflurane and placed belly-down with the head centered in a low-profile silicone anesthesia mask. A speaker was placed 12 cm to the left or right of the mouse facing directly toward the head. The same speaker and HiFiBerry sound card was used as in the behavioral training with the same calibration procedure, but the stimulus was a 0.1 ms click at either 60 or 70 dBA. The ABR measurement was repeated at least twice with the speaker on the left side and at least twice on the right. We measured the ABR on four separate occasions: immediately before hearing loss surgery, immediately after hearing loss surgery, and following the 6th and 7th post-operative behavioral sessions. The results were quite variable even within the same mouse, and so for each mouse we chose the post-operative measurement with the strongest and cleanest ABR, as assessed by an experimenter who was blinded to the type of hearing loss. Compared with ketamine/xylazine, isoflurane is known to increase the thresholds at which an ABR may be elicited, which may have increased variability in our results^103^.

We measured the ABR using an integrated circuit for multi-channel differential recording of biosignals (Texas Instruments ADS1299) and custom-built hardware to report the response to a computer. Regardless of the position of the speaker, the positive (i.e., non-inverting) and negative (inverting) electrodes were 30-gauge stainless steel needles placed in the skin just ventral to or just posterior to the intertragal notch. The ground electrode was the same type of needle placed in the skin above the neck or at the base of the tail. This system is designed to collect full-bandwidth data at a sampling rate of 16 kHz. We also measured the speaker voltage on another channel. We noted some cross-talk with the speaker voltage on the other channel of the amplifier and removed this by finding the best linear filter to predict neural voltage from speaker voltage and then removing this prediction from the neural signal. We post-processed the data by filtering it above 100 Hz, removing outlier trials (i.e., those in the top 5% of voltage excursion or standard deviation), and averaging approximately 750 repetitions.

The first peak in the ABR (“Wave 1”) originates from the auditory nerve^56^. Because the positive (non-inverting) electrode was on the left ear, Wave 1 is expected to be negative for sounds from the left and positive for sounds from the right. For presentation purposes we inverted the response to sounds from the left so that Wave 1 would in all cases be expected to be positive for a mouse with healthy hearing.

### Startle test

We assessed basic auditory-motor function before and after hearing loss using the acoustic startle reflex^64^. The same mice that were trained on sound-seeking were placed in a different behavioral chamber, a 17 cm by 18 cm by 27.5 cm rectangular prism constructed entirely of clear acrylic plastic and with a speaker mounted on both ends. Sounds were generated by the same type of Raspberry Pi, HiFiBerry sound card, and speaker as used in the sound-seeking task. The startle-eliciting sound was a 100 ms burst of broadband white noise at either 80 or 90 dB SPL. These two levels were interleaved, but we observed that the resulting behavior was fairly consistent so we pooled them. Continuous 60 dB white noise played throughout the experiment. The interval between sounds was a random time interval between 5 and 30 s. The entire procedure was repeated on five days: the third from last and the last behavioral session before surgery, and the first, fifth, and seventh sessions after surgery. The data presented here comprise only the last session before and the first and fifth sessions after surgery.

Before, during, and after sound presentation we took video of the mouse in the startle chamber at 640 × 480 resolution and 30 frames per second (White Matter e3vision). We tracked the position of the mouse’s head nose, limbs, trunk, and tail using the SLEAP algorithm. In this way we measured the “startle” response of the mouse after each sound, which is a full-body coordinated muscular contraction. Specifically, we measured the distance moved by each body part in pixels between each adjacent frame, averaged across all body parts, and aligned this movement to the onset of the sound.

### Data analysis

All data analysis was conducted in Python using the packages IPython^104^, pandas^105^, numpy^106^, scipy^107^, statsmodels^108^, and matplotlib^109^. We used SLEAP^65^ to measure body position in the startle test. The data and code necessary to regenerate the figures shown here will be shared upon publication of this manuscript.

In Fig. 7, we assessed statistical significance during learning as the effect of time in a one-way ANOVA; before and after hearing loss as the effect of surgery in a two-way ANOVA (pre/post x group) with a post-hoc t-test for each group; during recovery as the effect of time in a two-way ANOVA (day x group); and between groups during recovery as the effect of group in a two-way ANOVA (day x group) that only included the groups in question. All of these ANOVAs were repeated-measures because mouse identity was provided as a categorical regressor to the underlying linear model.

Throughout the manuscript, * indicates p < 0.05, ** p < 0.01, and *** p < 0.001.

### Supplementary Information

Supplementary Information.

## Data Availability

The datasets generated during the current study and the code necessary to generate the presented analyses and figures are available at https://github.com/Rodgers-PAC-Lab/Mai2024.
