# Supplementary material for: Sound-seeking before and after hearing loss in mice
Source: Sci Rep. 2024 Aug 19;14:19181. doi: 10.1038/s41598-024-67577-7 (PMC11333604; doi:10.1038/s41598-024-67577-7)
Supplement: Supplementary file 1 — Supplementary Information. [file 41598_2024_67577_MOESM1_ESM.pdf]

Supplemental Table 1. Mice used in this study.

Cohort	Group	Strain	Regulation
1	1A	CBA/CaJ;<br>C57BL6/J:CBA/J	deprivation
	1B	CBA/CaJ;<br>C57BL6/J:CBA/J	deprivation
	1C	CBA/CaJ	deprivation
2	2A	C57BL6/J:CBA/CaJ;<br>C57BL6/J:CBA/J	deprivation
	2B	CBA/CaJ	deprivation
3	3A	C57BL6/J:CBA/J	unpalatability
4	4A	C57BL6/J:CBA/CaJ	unpalatability
	4B	C57BL6/J:CBA/CaJ	unpalatability
	4C	C57BL6/J:CBA/CaJ	unpalatability
	4D	C57BL6/J:CBA/CaJ	unpalatability
5	5A	C57BL6/J:CBA/CaJ	unpalatability
	5B	C57BL6/J:CBA/CaJ	unpalatability

This table provides additional information about the mice used in this study, with cohort number and group names matching Table 1 in the main text. The column "Strain" indicates the strain of mouse used in each cohort, with a colon indicating an F1 hybrid of two strains. Groups 1A, 1B, and 2A comprised mice of different strains or hybrids, which are separated with a semicolon and a linebreak in the table. Mice were either purchased directly from Jackson Laboratories or bred in our animal facility. All breeder mice were purchased from Jackson Laboratories, with stock numbers 000664 (C57BL6/J), 000654 (CBA/CaJ), and 000656 (CBA/J).

The column "Regulation" indicates the water regulation paradigm used. "Deprivation" means that no water was available in the home cage. "Unpalatability" means that mice had ad libitum access in the home cage to water containing 1.5-3% citric acid, which lends a slightly sour flavor. In all cases, rewards during the behavioral task were fresh water from a water purification system (Millipore). Mice were monitored daily for health while on any form of water regulation.

The arena geometry was varied slightly throughout Cohorts 1, 2, and 3. For instance we tried different lengths and heights of the dividers between chambers, and we switched from mounting the speakers on the inside of the wall to a recessed holder instead. Additionally we tried longer inter-trial intervals up to 5 s, switched from one type of speaker (Multicomp Pro MCPCT-G5100-4139) to another (Almencla Ultrasonic Tweeter 2-inch Waterproof Piezo Horn), and refined our speaker calibration procedures. Cohorts 4 and 5 were trained with a consistent arena geometry, finalized speaker calibration, and an inter-trial interval of 1s.

In the main text, we described the "fixed parameters" and "variable parameters" task variants that were consistently used for Cohorts 4 and 5. As we were refining our procedures in Cohorts 1-3, we used the following slightly different task variants.

- Variant 1) Same as "fixed parameters" but with irregularity 1 ms instead of 31 ms
- Variant 2) Same as "variable parameters" but with repetition rate ranging from 1-12 Hz instead of 1-10 Hz, center frequency ranging from 5-18 kHz instead of 5-15 kHz, and sound level ranging from 50-90 dB SPL instead of 65-90 dB SPL;
- Variant 3) Same as "fixed parameters" but with repetition rate fixed at 10 Hz instead of 4 Hz, irregularity fixed at 10 ms instead of 31 ms, level fixed at 80 dB SPL instead of 70 dB SPL, and bandwidth ranging from 3-9 kHz instead of fixed at 3 kHz
- Variant 4) same as "fixed parameters" but with irregularity ranging from 1-316 ms
- Variant 5) same as "fixed parameters" but with repetition rate fixed at 6 Hz and sound level fixed at 80 dB SPL

Mice in Cohort 1 were trained on variants 1-4. Mice in Cohort 2 were trained on variants 1 and 5. Mice in Cohort 3 were trained on variant 5. After these cohorts, we adopted the consistent parameters described in the main text.

Supplemental Figure 1. Learning across each cohort

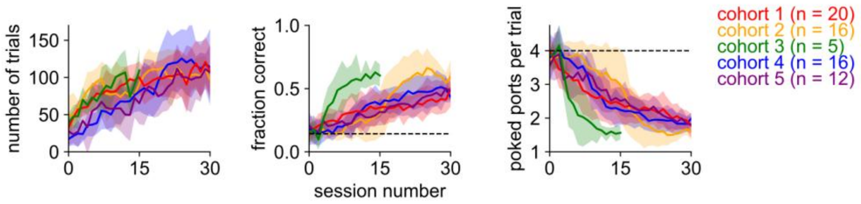

Learning was variable between cohorts of mice but all cohorts reached a roughly similar level of final performance.

Supplemental Figure 2. Auditory brainstem response (ABR) after hearing loss

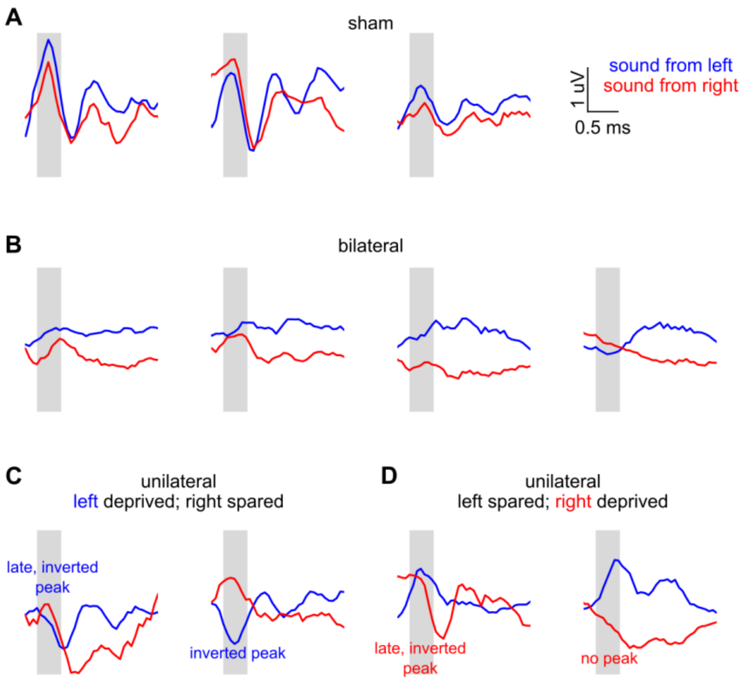

A. Each ABR (i.e. pair of red and blue traces) is for a single mouse. Panel A shows the ABR for three mice following sham hearing loss surgery in response to a click from left (blue) or right (red). Gray shaded box indicates 2.0 - 2.3 ms after the click, the expected time of the first peak ("Wave 1"). To calculate the ABR, we measured a differential voltage between the left and right ear and averaged over about 1000 clicks. The ABR signal has been inverted for sounds from the left, which in a healthy mouse should make Wave 1 positive in both the red and blue traces. All three mice show strong, positive peaks within the shaded box for sounds from both sides.

B. ABR following bilateral hearing loss. Wave 1 is attenuated, delayed, or abolished.

C. ABR following unilateral (left) hearing loss. Wave 1 is delayed or inverted (negative) for sounds from the left (blue). Because of the differential recording procedure, the inversion likely represents a positive response at the other (spared) ear.

D. ABR following unilateral (right) hearing loss. Wave 1 is delayed or inverted (negative) for sounds from the right (red).

Supplemental Figure 3. Inclusion of returns to the previously rewarded port does not substantially affect results.

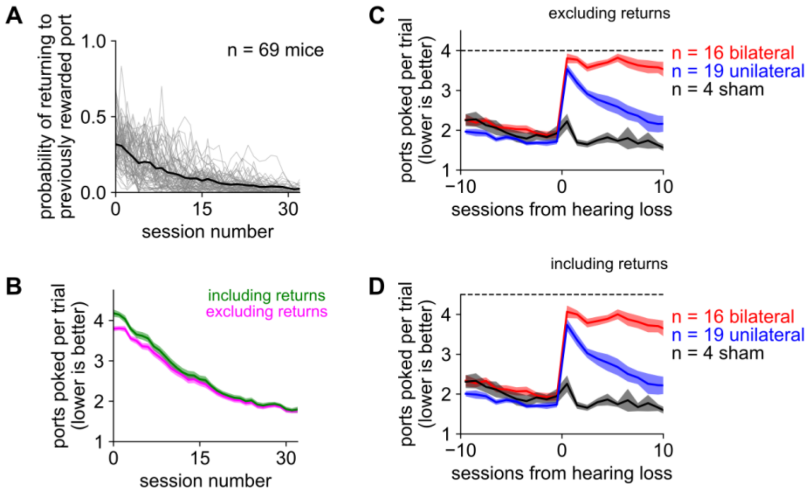

A. Probability of returning the previously rewarded port. Gray lines: individual mice. Black line: average.

B. Performance over learning, measured as mean number of ports poked per trial. Including returns (green) only slightly changes the learning curve compared to excluding returns (pink). Figures in the main text always exclude returns. Throughout this figure, error bars: SEM.

C. Performance before and after hearing loss. This panel excludes returns, and is copied from Fig 4C in the main text as a point of comparison for panel D.

D. Performance before and after hearing loss when returns are included. Results are similar to panel C, which excludes returns. Note that the chance rate is slightly higher if returns are included, because the number of ports poked now ranges from 1-8 instead of 1-7.
